# Changing from a two-tiered to a one-tiered trauma team activation protocol: a before–after observational cohort study investigating the clinical impact of undertriage

**DOI:** 10.1007/s00068-021-01696-y

**Published:** 2021-05-22

**Authors:** Kenneth Thorsen, Jon Kristian Narvestad, Kjell Egil Tjosevik, Johannes Wiik Larsen, Kjetil Søreide

**Affiliations:** 1 Section for Traumatology; Surgical Clinic, Stavanger University Hospital, Stavanger, Norway; 2 Department of Gastrointestinal Surgery, Stavanger University Hospital, PO Box 8100, 4068 Stavanger, Norway; 3 Department of Emergency Medicine, Stavanger University Hospital, Stavanger, Norway; 4 Department of Clinical Medicine, University of Bergen, Bergen, Norway

**Keywords:** Trauma, Trauma team, Trauma team activation protocol, Mortality, Injury severity

## Abstract

**Background:**

The aim of this study was to compare the effect of the change in TTA protocol from a two-tier to one-tier, with focus on undertriage and mortality**.**

**Material and methods:**

A before–after observational cohort study based on data extracted from the Stavanger University Hospital Trauma registry in the transition period from two-tier to a one-tier TTA protocol over two consecutive 1-year periods (2017–2018). Comparative analysis was done between the two time-periods for descriptive characteristics and outcomes. The main outcomes of interest were undertriage and mortality.

**Results:**

During the study period 1234 patients were included in the registry, of which 721 (58%) were in the two-tier and 513 (42%) in the one-tier group. About one in five patients (224/1234) were severely injured (ISS > 15).

Median age was 39 in the two-tier period and 43 years in the one-tier period (*p* = 0.229). Median ISS was 5 for the two-tier period vs 9, in the one-tier period (*p* = 0.001). The undertriage of severely injured patients in the two-tier period was 18/122 (15%), compared to 31/102 (30%) of patients in the one-tier period (OR = 2.5; 95% CI 1.8–4.52). Overall mortality increased significantly between the two TTA protocols, from 2.5 to 4.7% (*p* = 0.033), OR 0.51 (0.28–0.96)

**Conclusion:**

A protocol change from two-tiered TTA to one-tiered TTA increased the undertriage in our trauma system. A two-tiered TTA may be beneficial for better patient care.

## Introduction

Trauma is a leading cause of death and disability for people < 45 years of age worldwide, including the Nordic countries [1–3]. To minimise the consequences of trauma, optimal care in all stages of the trauma management are essential [4]. Protocols have been put in place to ensure trauma team activation (TTA) for patients who are severely injured or believed to be at risk of serious injury. The jeopardy of any protocol and its criteria is the risk of either over- or under-triage [5]. When a trauma patient with a major injury, defined as ISS > 15, is not met by a trauma team, the patient is undertriaged [5]. Undertriage may be the result of too stringent criteria for TTA, failure to recognise the criteria in the prehospital field or due to an inappropriate staffed team. Such undertriage may be associated with longer time to diagnosis, longer time to lifesaving interventions and higher risk of death [6, 7]. To avoid undertriage, criteria are set to allow for a certain degree of overtriage. An undertriage of < 5% and an overtriage of 25–50% has been proposed as acceptable by the American College of Surgeons [8].

However, several studies from Norway has described considerable higher undertriage than the accepted 5% [5, 9, 10] and also considerable higher overtriage than accepted, of about 80% [10].

A previous report found a favourable effect of revising TTA to a two-tiered team protocol, with a regular full 13-member team activation for all major trauma and a reduced team given specific criteria for TTA [5]. The two-tier protocol led to a significant reduction in undertriage from 28.4 to 19.1% after system revision. However, with mandated implementation of a national trauma plan, the institutional TTA protocol was returned to a one-tier TTA, without good data to support this decision. Hence, the current study was designed to evaluate the effect of going back to a one-tier protocol from the previously reported two-tier TTA.

The aim of this study was to compare two time periods before and after TTA protocol change with focus on the impact on undertriage, characteristics of undertriaged patients and mortality.

## Material and methods

### Ethics

This study was accepted as a quality improvement project by the personal data officer in Stavanger University Hospital. The SUH trauma registry also has approval from the personal data officer as a quality registry.

### Study design

A retrospective, consecutive, observational cohort based on a before-and-after evaluation of two time periods, the “before-period” with a two-tier system compared the “after-period” following system revision back to a one-tier TTA. The study is performed and reported according to the STROBE guidelines where applicable [11].

### Study population

All data were extracted from the Stavanger University Trauma registry from 2017 to 2018.

For the undertriaged patients the electronic patient journals were also investigated with regards to identifying potential relative trauma criteria present in the patient chart.

The Stavanger region serves a population of about 370.000 people and receives injured patients from about 500.000 inhabitants in a wider catchment area. Annually, about 550 patients are admitted to SUH for suspected or potentially severe trauma and about one in five of the admitted patients have an ISS > 15. Since January 1, 2004 a formal trauma registry has been in place, including all patients admitted with a trauma alarm and patients with an ISS ≥ 9 not receiving a trauma alarm from pre-set criteria [12].

Patients admitted to the emergency department without trauma team activation but who are found to have an Injury Severity Score (ISS) > 9 on diagnostic screening or, have a penetrating injury to the head/neck/torso proximal to the elbow or knee, head injury with Abbreviated Injury Scale (AIS) ≥ 3 or ≥ 2 proximal long bone fractures are registered in the trauma registry by the trauma registrars. Patients with mild head injuries or isolated femoral neck fractures are not routinely included in the registry.

Patients with a trauma alarm found to have only a medical issue were excluded. Patients found dead at scene were also excluded.

### Definition of time periods

The before period was set from January 1st to December 31st, 2017. In this period a two-tier TTA protocol was used, as previously described [5]. A full TTA (13 team members) was initiated after prespecified criteria. A reduced TTA (surgeon on-call + ED staff) was initiated according to a limited set of criterias, described previously [5].

The after period was from January 1st to December 31st, 2018. In this period a one-tier protocol with only a full TTA, was applied.

### Definitions

Severe injuries were defined as an injury severity score (ISS) > 15 [13]. The Association for the Advancement of Automotive Medicine—Abbreviated Injury Scale 1990 revision, update 98 (AIS 98) [14] was used, since this version derived the ISS > 15 threshold for defining major trauma.

#### Over- and under-triage

Overtriage is defined as any TTA for trauma patients with an ISS ≤ 15. Undertriage is defined as the lack of TTA for a trauma patient with a severe injury defined by an ISS > 15 [8].

### Statistics

Statistical analysis was performed with The Statistical Package for Social Sciences, SPSS® version 26 for Mac (IBM, Armonk, New York, USA).

Descriptive analysis was performed with Chi-squared test or Fisher’s exact, where appropriate for categorical variables and Kruskal–Wallis test or Mann–Whitney *U* test for continuous variables.

All tests are two-sided and a *p* value of < 0.05 deemed statistically significant.

## Results

**Fig. 1:**
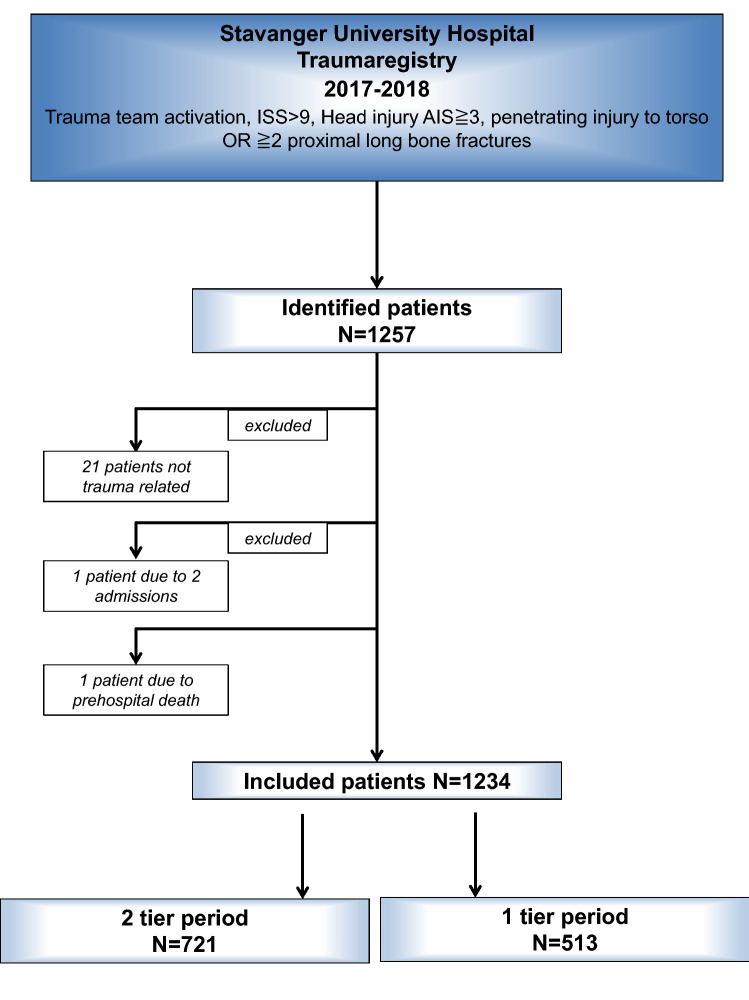
Patient flow chart

The patient inclusion criteria are shown in the flow chart (Fig. 1), resulting in 1234 patients in total, divided in 721 patients in the two-tier period versus 513 patients in the one-tier period. Patient characteristics comparing the one-tier versus two-tier period are shown in Table 1 and composition of the trauma team in Table 2.

**Table 1:** Comparison of patients in the 2-tier vs 1-tier TTA cohorts

Category	Total	2-tier TTA	1-tier TTA	*P*	OR (95% CI)
Period, *n* (%)	1234 (100%)	721 (58%)	513 (38%)	< 0.001	
Gender					
Female, *n* (%)	413 (33.5%)	233 (32.2%)	180 (35.2%)		
Male, *n* (%)	821 (66.5%)	488 (67.8%)	333 (64.8%)	0.272	0.9 (0.7–1.1)
Age (years)					
Median, range	40 (0–106)	39 (0–106)	43 (0–99)	0.229	
Elderly (≥ 60 years), *n* (%)	315	170	145	0.068	1.3 (1.0–1.6)
Injury severity					
ISS, median (range)	5 (1–75)	5 (1–75)	9 (1–75)	< 0.001	
Severe injury (ISS > 15), *n* (%)	224 (18.2%)	122 (16.9%)	102 (19.9%)	0.183	1.2 (0.9–1.6)
NISS median (range)	6 (1–75)	6 (1–75)	9 (1–75)	0.001	
Severe injury (NISS > 15), *n* (%)	314 (25.4%)	170 (23.6%)	144 (28%)	0.074	1.3 (1.0–1.6)
Trauma mechanism					
Blunt	1198 (97%)	689 (95.6%)	489 (95.3%)	0.642	1.1 (0.7–1.9)
Penetrating	62 (5%)	34 (4.7%)	28 (5.4%)		0.9 (0.5–1.4)
Mechanism of injury					
Traffic, *n* (%)	544 (44%)	331 (46%)	213 (41%)	0.121	1.2 (1.0–1.5)
Fall, *n* (%)	579 (47%)	366 (51%)	213 (42%)	0.001	1.5 (1.2–1.8)
Violence, *n* (%)	64 (5.2%)	35 (5%)	29 (6%)	0.809	0.9 (0.5–1.4)

**Table 2:** Trauma team composition

Full team	Reduced team
Team leader surgeon	Team leader surgeon
Orthopaedic surgeon	Orthopaedic surgeon
Anaesthetist	Two ED nurses
Nurse anaesthetist	
Radiologist	
Two radiographers	
Three ED nurses	
Theatre nurse	
Laboratory technician	
Orderly	

Median ISS was 5 for the two-tier period vs 9, in the one-tier period (*p* = 0.001).

A total of 224 patients had an ISS > 15 and 49/224 (21.9%) were undertriaged.

Of the patients with ISS > 15, 18/122 (14.8%) of patients were undertriaged in the two-tier period compared to 31/102 (30.4%) in the one tier period (*p* = 0.002, OR 2.5 (95% CI 1.4–4.5).

The under- and overtriage per cohort, as well as the associated mortality is given in Table 3.

**Table 3:** Comparison of outcomes between the two-tier vs one-tier TTA cohorts

Category	Total	Two-tier TTA	One-tier TTA	*p*	OR (95% CI)
Patients, *n* (%)	1234 (100%)	721 (58%)	513 (38%)		
Undertriage	49/224 (21.9%)	18/122 (14.8%)	31/102 (30.4%)	0.002	2.5 (1.4–4.5)
Overtriage	928/1101 (84.3%)	562/665 (84.5%)	366/436 (84.0%)	0.008	0.7 (0.5–0.9)
Correct triage	257/1234 (20.8%)	141/721 (19.6%)	116/513 (22.6%)	0.98	1.0 (0.7–1.4)
Mortality, *n* (%)	42 (3.4%)	18 (2.5%)	24 (4.7%)	0.033	0.5 (0.3–1.0)

All the undertriaged patients had a blunt trauma mechanism and no difference in undertriage between gender was encountered (*p* = 0.899).

Undertriaged patients were further characterised by a median age of 66, while those who were not undertriaged had a median age of 38 (*p* < 0.001).

When comparing those undertriaged with patients not undertriaged, the patients > 65 years of age were more likely to be undertriaged (*p* < 0.001), OR 4.5 (95% CI 2.5–8.0) constituting 25/49 (51%) of the undertriaged patients, while the age group > 65 years only constituted 249/1234 (20%) of the total trauma population. Univariate analyses of associations with undertriage is shown in Table 4.

**Table 4:** Univariate analyses of undertriaged patients

	Undertriaged patients (*N* = 49)	Not undertriaged patients (*N* = 1185)	*p* value	OR (95% CI)
Median age (IQR)	66 (52–83)	38 (22–58)	< 0.001	
Median GCS(IQR)	15 (14–15)	15 (14–15)	0.747	
Sex				
Male	33 (67%)	787 (66%)		
Female	16 (33%)	397 (34%)	0.899	1.04 (0.57–1.91)
Age				
≤ 65 years	24/983 (2.4%)	959/983 (97.6%)		
> 65 years	25/249 (10%)	224/249 (90.0%)	< 0.001	4.5 (2.5–8.0)
Transport injury				
Yes	8 (16.3%)	537 (45.3%)		
No	41 (83.7%)	646 (54.7%)	< 0.001	4.26 (1.98–9.17)
Fall injury				
Yes	36 (73.5%)	542 (45.7%)		
No	13 (26.5%)	636 (54.3%)	0.001	0.31 (0–1–0.59)
Blunt	49/1178 (4.2%)	1128/1178 (95.8%)		
Penetrating	0/56 (0%)	56/56 (100%)	0.103	0.96 (0.95–0.97)
30-day mortality	8 (16.3%)	34 (2.9%)	< 0.001	6.2 (2.7–14.1)
2-tier period	18/721 (2.5%)	703/721 (97.5%)		
1-tier period	31/513 (6.0%)	482/513 (94%)	0.002	2.5 (1.4–4.5)

Most of the undertriaged patients had a relative criteria present. Key elements of every undertriaged patient is shown in Table 5.

**Table 5:** Undertriaged patients at Stavanger University hospital in the (a) before period (2017), (b) after period (2018)

Patient	Age	sex	Injury mechanism	Absolute criteria	Relative criteria	ISS	Dominating injury	30-day mortality
(a)								
1	81	Female	Low-energy fall	No	Yes	29	Subdural hematoma	Yes
2	21	Male	Fall from skateboard	No	Yes	17	Open tibial fracture	No
3	38	Male	Low-energy fall	No	No	17	Epidural/subdural hematoma	No
4	52	Male	Low-energy fall	No	Yes	29	Subdural hematoma	No
5	65	Female	Fall	No	Yes	41	Liverlaceration/subarachnoidal bleeding	No
6	62	Male	Bicycle	No	Yes	18	Subarachnoidal bleeding	No
7	92	Male	Low-energy fall	No	Yes	17	Subdural hematoma	No
8	69	Male	Fall	No	Yes	16	Subarachnoidal/subdural bleeding	No
9	90	Female	Fall in stairs	Yes	Yes	26	Subdural hematoma/costa fractures	Yes
10	91	Male	Low-energy fall	No	Yes	16	Hemothorax/multiple costa fractures	No
11	77	Male	Low-energy fall	Yes	Yes	24	Subdural hematoma/subarachnoidal bleeding/skull fracture	No
12	89	Male	Fall in stairs	No	Yes	17	Subdural bleeding	No
13	61	Male	Low-energy fall	No	Yes	17	Multiple costa fractures	No
14	51	Male	Bicycle	No	No	17	Subdural hematoma, C4 fracture	No
15	67	Male	Low-energy fall	No	Yes	17	Subdural hematoma	Yes
16	60	Male	Low-energy fall	Yes	Yes	25	Bilateral subdural hematoma	No
17	57	Male	Motorcycle	No	No	16	Large pneumothorax, costafractures	No
18	63	Female	Low-energy fall	No	Yes	17	Subdural hematoma	No
(b)								
1	80	Female	Low-energy fall	No	Yes	24	Subdural hematoma	No
2	76	Female	Low-energy fall	No	Yes	17	Intracerebral bleeding	No
3	98	Female	Low-energy fall	No	Yes	21	Subdural hematoma	No
4	3	Female	Hit by heavy object	No	Yes	16	Skull fracture, cerebellar bleeding	No
5	92	Male	Low-energy fall	No	Yes	16	Subdural hematoma	No
6	13	Female	Fall from horse	No	Yes	17	Liver laceration grade 4–5	No
7	7	Male	Fall	No	Yes	16	Spleen injury grade 4	No
8	93	Female	Low-energy fall	No	Yes	17	Subarachnoidal/subdural bleeding	Yes
9	44	Male	Fall	No	No	16	Subdural hematoma/skull fracture	No
10	50	Male	MVA	No	Yes	19	Multiple costa fractures	No
11	59	Male	Hit by heavy object	No	Yes	17	Subarachnoidal bleeding	No
12	80	Male	Low-energy fall	No	Yes	10	Small subdural hematoma	No
13	6	Female	Fall	No	Yes	18	Spleen injury grade 3 /pneumothorax	No
14	48	Male	Low-energy fall	No	No	17	Subarachnoidal/subdural bleeding	No
15	48	Male	Bicyle	No	No	17	Subarachnoidal/subdural bleeding	No
16	83	Female	Low-energy fall	No	Yes	26	Subdural hematoma	Yes
17	66	Male	Fall	Yes	Yes	16	Multiple costa fractures	No
18	38	Female	Bicycle	No	No	17	Skull fracture	No
19	72	Male	Low-energy fall	No	Yes	17	Subarachnoidal/subdural bleeding	No
20	60	Male	Bicycle	No	Yes	21	Subdural hematoma	No
21	85	Male	Low-energy fall	No	Yes	20	Multiple costa fractures	No
22	82	Male	Low-energy fall	No	Yes	17	Subarachnoidal/subdural bleeding	No
23	86	Male	Low-energy fall	No	Yes	17	Subdural bleeding	No
24	25	Male	Sporting injury	Yes	No	25	Aortic laceration	Yes
25	59	Male	Low-energy fall	No	Yes	17	Subarachnoidal/subdural bleeding	No
26	60	Male	Low-energy fall	No	Yes	16	Multiple costa fractures/pneumothorax	No
27	72	Male	Low-energy fall	No	Yes	21	Subdural hematoma	No
28	80	Female	Low-energy fall	No	Yes	24	Neck injury/spinal cord	No
29	85	Female	Low-energy fall	No	Yes	25	Subdural hematoma	Yes
30	79	Female	Fall in stairs	No	Yes	24	C7 fracture, medulla contusion, facial fracture	Yes
31	59	Male	Fall in stairs	Yes	Yes	21	Subdural hematoma/skull fracture/costa fractures	No

It was more likely for patients with fall as trauma mechanism compared to patients without fall as traumamechanism to be undertriaged (*p* = 0.001), OR 0.31 (95% CI 0.16–0.59).

When comparing undertriaged patients with other trauma mechanism than transport injury, they were more likely to be undertriaged than patients with transport injury as trauma mechanism (< 0.001), OR 4.26 (95% CI 1.98–9.17).

Patients that were undertriaged were more likely to succumb within 30 days, than patients who were not undertriaged (*p* < 0.001, OR 0.16 (95% CI 0.07–0.36).

The criteria for trauma team activation is shown in Table 6 for the before period and in Table 7 for the after period.

**Table 6:** Trauma team criteria in the before period

Full trauma team	Reduced trauma team
RTS ≤ 11	Age < 60 years
GCS < 14	Age < 6 years
Respiration rate < 9/min	Severe comorbidity (COPD, heart failure etc.)
Respiration rate > 25/min	Pregnancy
Spo2 < 90%	Increased risk of bleeding (anticoagulant drugs, coagulopathy)
Intubated/attempted intubation	
Obvious massive haemorrhage	Co-passenger killed
Systolic blood pressure < 90 mmHg	Entrapped person
	Person ejected from vehicle/motorcycle
Facial injury with risk for airway obstruction	Pedestrian, cyclist run down at > 30 km/h or thrown up in the air
Flail chest	Collision speed > 50 km/h
Suspected pneumothorax	Deformed vehicle compartment
Stab or gunshot wound proximal to knee or elbow	Airbag set off
Suspected pelvic fracture	Vehicle roll-over
Crushed, mangled or amputated extremity	
Two or more long bone fractures	Fall > 5 m (adults)
Open fracture with ongoing haemorrhage	Fall > 3 m (children)
Open skull fracture or impression fracture	Interhospital transfer and < 24 h since time of injury
Suspected spinal cord injury	
Burn injury (≥ grade II) > 15% total body surface area	
Accident with several severely injured (suspected or confirmed) patients	
Upgrade to full trauma team	
When two or more criteria for reduced trauma team are fulfilled	
When reduced trauma team finds a perceived stable patient to be unstable

**Table 7:** National trauma team criteria in the after period

Trauma team
Vital functions
Respiration rate < 10/min
Respiration rate > 29/min (or need of ventilatory support)
Spo_2_ < 90% without O2
Systolic blood pressure < 90 mmHg
Pulse < 130/min
Severe hypothermia without normal circulation
Anatomy
Facial injury with risk for airway obstruction
Open skull fracture or impression fracture
Penetrating injury to face, neck, torso or extremities proximal to elbow or knee
Strong thoracic pain (suspicion of multiple costa fractures)
Large external bleeding
Large crush injuries
Two or several large fractures
Strong pelvic pain (suspicion of pelvic fracture)
Suspected spinal cord injury
Injury to two body regions (head/neck/thoracic/abdominal/pelvic/spine/femur)
Burn injury (≥ grade II) > 15% total body surface area (children 10%) or inhalation injury
Mechanism of injury
Collision speed > 50 km/h without seat belt or airbag not released
Vehicle roll-over
Entrapped person in vehicle
Person ejected from vehicle/motorcycle
Cyclist or pedestrian hit by motor vehicle
Fall > 5 m (adults), Fall > 3 m (children)
IF any of the criterias below are present a lower threshold for TTA is mandated
Age > 60 years
Age < 5 years
Severe comorbidity (COPD, heart failure etc.)
Pregnancy > 20 weeks
Increased risk of bleeding (anticoagulant drugs, coagulopathy)
Intoxication

There seemed to be somewhat of a cluster of undertriage during winter, with the four winter months: December–March represented 27/50 (54%) of the undertriaged patients.

After changing from two-tier to one-tier approach the annual admittance rate of trauma patients decreased significantly. The total number of patients with ISS > 15 was also lower, but not significantly. Median ISS in the two-tier period was 13 for patients with no TTA (Neither full nor reduced) versus five for those with a TTA (*p* < 0.001).

In the one-tier period patients with no TTA had a median ISS of 14 versus five for those with TTA (p < 0.001).

Including only patients with an ISS > 15, the mortality was 13.7% (14/102) in the one-tier period versus 10.7% (13/122) in the two-tier period (*p* = 0.482).

Including only patients with an ISS ≤ 15, the mortality was 2.8% (11/393) in the one-tier period versus 0.08% (5/599) in the two-tier period (*p* = 0.019).

## Discussion

In the current study, a change in protocol from two-tiered TTA to one-tiered TTA caused a significant increase in undertriage from 15 to 30%, with an odds ratio of 2.5 for undertriage in the after period. The overall mortality increased, but this has to be considered in the light of substantially lower volume of trauma patients admitted in the one-tier period. In addition, due to the change in protocol and lower admittance rate, the patients in the after period had a statistically significant higher median ISS. However, the mortality for the group with ISS > 15 also increased, but not statistically significant. As such we believe the increase in mortality to be explained first and foremost by the lower denominator in the after period.

The undertriaged patients were dominated by patients > 65 years of age, with fall as trauma mechanism and head injury as the dominating injury.

The patients that did not receive a TTA had a significantly higher ISS than patients who did receive a TTA. This can be seen as a paradox, yet is, at least to an extent, a function of the current TTA criteria and method of identifying undertriaged patients retrospectively. Further, the patients who did not receive any TTA were significantly older than patients who did receive a TTA. Patients with fall as trauma mechanism were more likely not to receive a TTA than patients with other mechanisms than fall during both periods.

Several other studies have found similar results, with older patients with low-energy falls being undertriaged [5, 9, 15].There may be several reasons for this. The undertriaged patients in both the before and after period were dominated by geriatric patients with low-energy falls and head injuries as the dominating injury. Most of the undertriaged patients were found to have a relative criterion that would yield a reduced team in the before period, while this did not yield a TTA in the after period, but stated “a lower threshold for TTA present if any of the criteria present”. Hence, the lack of a reduced team seems to have given the prehospital personnel a higher threshold for trauma alarm set-off, understanding the consequences overtriage with a full trauma team puts on hospital resources. In some of the cases the trauma criteria were not met, even though the patient later is found to have an ISS > 15, the corresponding physiologic parameters are not always present. And second, there might exist a prehospital bias to low-energy falls that misleads the prehospital team. The relative criteria in the before period yielded a reduced team, while these criterias are specified as “if any of these criteria are present (see Table 7), a lower threshold for TTA is present” in the current national triage criteria. Ironically, earlier attempts to improve triage precision reduced undertriage from 28 to 19% after implementing a two-tiered TTA [5]. These results question both the one-tier model and the trauma criterias used for the Norwegian trauma population. However, several studies from Norway have described considerable higher undertriage than the accepted 5% [5, 9, 10, 16] and also considerable higher overtriage than accepted, of about 80% [10] using a one tier TTA model. A fresh Dutch study estimated considerable improvements in triage precision after identifying the most optimal triage criterias in their own trauma population [15].

Efforts have been made elsewhere to address patients > 65 years of age with low--energy falls, with expedited teams specifically addressed for this group. One study found to decrease length of stay, but with no improvement in mortality after implementing an expedited team [17]. If we are to provide a decent and optimal care for older patients with low injury falls, a change in the trauma system may be necessary. It appears that the trauma criterias in use based on the field triage criterias does not function optimally for this patient group. As a consequence of the findings in this study, we are introducing a specific geriatric trauma team in SUH, that will seek to address and improve both undertriage and trauma care for the geriatric trauma patients in SUH, specifically for the older patients with low-energy falls and suspected head injury.

Overtriage does not impact the trauma patient negatively per se but may lead to trauma team fatigue by a high rate of “false alerts” or a feeling of “cry wolf” to set alarm criteria. Importantly, other potentially sick patients may suffer in the lack of allocated resources when competing for the same personnel and time. An overtriage of > 80% as seen in both periods in this study is too high and mandates scrutiny. However, to reduce undertriage one has to accept a certain degree of overtriage, where 25–50% has previously been described as acceptable by the American college of surgeons.

We question the generic recommendation of one-tiered TTA as recommended in the Norwegian national trauma guidelines and, a change in TTA protocol and/or TTA criteria that is fitted to the need of any specific hospital trauma volume and population seems warranted.

The tendency to a cluster of undertriaged patients admitted in the four winter months was somewhat surprising. We do not have an explanation for this and can only speculate about this finding. It may be just a coincidence since only two years were analysed. But if the finding represents a real cluster, perhaps less sun and potentially vitamin D deficiency can contribute and explain to some degree why there seemed to be a cluster of undertriaged patients (geriatric patients with low-energy falls) during the winter months.

Some limitations should be addressed. This is a registry study with its implied strengths and weaknesses. Also, since we chose to directly compare only the year before and after, this could potentially affect the study power yielding potential type II errors due to too low patient volume. It should also be kept in mind that the findings in this single centre study are not necessarily generalizable for other regions and protocols.

## Conclusion

After changing protocol from two-tiered TTA to one-tiered TTA, the undertriage doubled.

Undertriage was associated with falls and age > 65 years with head injury as dominating injury. An increase in the overall mortality was also observed, but needs to be mirrored by the decrease in the overall trauma admittance rate, yielding a lower denominator in the one-tier period.
